# The Bacterial Microbiome Associated With Arid Biocrusts and the Biogeochemical Influence of Biocrusts Upon the Underlying Soil

**DOI:** 10.3389/fmicb.2019.02143

**Published:** 2019-09-23

**Authors:** Benjamin Moreira-Grez, Kang Tam, Adam T. Cross, Jean W. H. Yong, Deepak Kumaresan, Paul Nevill, Mark Farrell, Andrew S. Whiteley

**Affiliations:** ^1^UWA School of Agriculture and Environment, Faculty of Science, The University of Western Australia, Perth, WA, Australia; ^2^Centre for Mine Site Restoration, Department of Environment and Agriculture, Curtin University, Perth, WA, Australia; ^3^School of Biological Sciences, Faculty of Science, The University of Western Australia, Perth, WA, Australia; ^4^Department of Biosystems and Technology, Swedish University of Agricultural Sciences, Alnarp, Sweden; ^5^School of Biological Sciences, Queen’s University Belfast, Belfast, United Kingdom; ^6^CSIRO Agriculture and Food, Urrbrae, SA, Australia

**Keywords:** biological soil crust, biocrust, 16S rRNA, microbial communities, δ^15^N, semi-arid environment

## Abstract

Biocrusts are aggregated crusts that exist on the soil surface of arid environments. They are complex microbial communities comprised of cyanobacteria, lichens, mosses, algae and fungi. Recently, biocrusts have gained significant attention due to their ubiquitous distribution and likely important ecological roles, including soil stabilization, soil moisture retention, carbon (C) and nitrogen (N) fixation, as well as microbial engineers for semi-arid ecosystem restoration. Here, we collected three co-occurring types of biocrust (Cyanobacterial crust, Crustose lichen, and Foliose lichen) and their underlying soil from arid zones within Western Australia. Bacterial microbiome composition was determined through 16S rRNA gene amplicon sequencing to assess the extent of microbiome selection within the crusts versus underlying soil and biogeochemical measures performed to determine whether the crusts had significant impact upon the underlying soil for nutrient input. We determined that the bacterial communities of native biocrusts are distinct from those in their underlying soil, where dominant bacterial taxa differed according to crust morphologies. δ^15^N revealed that N-fixation appeared most evident in Foliose lichen crust (1.73 ± 1.04‰). Consequently, depending upon the crust type, biocrusts contained higher concentrations of organic C (2 to 50 times), total N (4 to 16 times) and available ammonium (2 to 4 times), though this enrichment did not extend to the soils underneath them. These findings demonstrate that biocrust communities are seemingly islands of biological activity in an arid landscape, uniquely different from their surrounding and underlying soil.

## Introduction

Biological soil crusts (“biocrusts” hereafter) are complex communities comprised of cyanobacteria, lichens, bryophytes, fungi, and algae found on soil surfaces and are common and integral components of arid and semi-arid ecosystems, where plant cover is often sparse (Belnap et al., 2001; Rodriguez-Caballero et al., 2018). Biocrusts can cover up to 70% of the soil surface in desert areas (Yeager et al., 2004) and are estimated to cover over 12% of global terrestrial land surfaces (almost 18 million km^2^; Rodriguez-Caballero et al., 2018). Due to their ecological and microbial characteristics, aligned with their ubiquitous occurrence in arid environments, biocrusts have emerged as an important and emerging research focus in recent decades (Belnap and Lange, 2001; Belnap and Weber, 2013; Bowker et al., 2018), addressing many research themes, including stress survival, ecological assembly in harsh environments and biogeochemical influence on their surrounding soils.

Biocrusts thrives in severe arid environments, performing a variety of significant ecosystem services (Belnap, 2003), including carbon (C) and nitrogen (N) fixation (Evans and Ehleringer, 1993; Belnap, 2002), improving soil particle aggregation (Eldridge and Leys, 2003), reducing soil erosion (Chamizo et al., 2017), regulating soil hydrology (Belnap et al., 2013) and the promotion of vascular plant development in some situations (Ghiloufi et al., 2016; see Zhang et al., 2016 for review). Elbert et al. (2012) estimated mean global carbon content within biocrusts to be approximately 4.9 Pg, representing one percent of terrestrial carbon in vegetation. Furthermore, biocrust-driven N-fixation is a major source of nitrogen input into the arid environment (Belnap, 2002). Indeed, it has been estimated that biocrusts may be responsible for nearly 46% of global N-fixation activity in terrestrial ecosystems (24.30 Tg yr^–1^ N; Elbert et al., 2012; Rodriguez-Caballero et al., 2018). Furthermore, the release of nitrogenous products (e.g., ammonium, nitrate, amides, peptides, and amino acids) has been observed in some cyanobacteria and lichens that make up these biocrusts (see Barger et al., 2016 for review). Millbank (1978, 1982) and Silvester et al. (1996) found labeled ^15^N in the extracellular environment of N-fixing cyanobacteria, showing that fixed N may be released into the surrounding soil environment, representing a potential pathway for N enrichment in topsoil directly underlying biocrusts and a vehicle to initiate soil N cycles for future plant colonization.

A significant proportion of Western Australia is arid or semi-arid land with a wide distribution of biocrusts, yet almost nothing is known about them within this significant land mass. This is perhaps best illustrated in a recent study by Rodriguez-Caballero et al. (2018), where over 500 “biocrust” publications were analyzed to predict global biocrust coverage. Whilst over half of Western Australia’s surface was projected to contain biocrusts, no actual data was used to originated from the region. Further, microbial characterization of biocrusts is crucial as many communities were found to be geographically distinct (Strauss et al., 2012), which may have implications for the biogeographical spread of functional capabilities (e.g., N-fixation). Utterly, a better understanding of these cryptic communities in terms of composition and functionality can directly beneficiate by creating next-generation, holistic restoration protocols for semi-arid landscapes, both in WA and worldwide.

In this study, three distinct biocrust (i.e., Cyanobacterial crust, Crustose lichen, and Foliose lichen) communities were sampled from undisturbed vegetation sites in the Midwest region of Western Australia. High-throughput sequencing of 16S rRNA gene and stable isotope analysis were employed to address the following questions: (1) What are the microbial communities within native biocrusts and how do they differ between crust types and their underlying soil? (2) What is the N-fixing potential within different biocrusts? (3) How does N content differ between biocrusts in relation to N-fixation? And (4) whether the presence of N-fixing biocrusts increase N content in underlying soil? These analyses are required to fill fundamental knowledge gaps of the global distribution and ecology of biocrusts and to assess whether biocrusts display characteristics that could likely be used in ecological restoration in harsh arid environments. We conclude that different biocrust types display unique microbial characteristics on both phylogenetic and functional (i.e., N-fixation) levels. Furthermore, each crust type displays characteristics which are distinct from bare soil and the soil underneath biocrust, suggesting they exist as “ecological islands” within the landscape. For the first time, these data shed light on the differentiation of different types of biocrusts within the West Australian landscape and their interactions with the soil surrounding them.

## Materials and Methods

### Study Site and Sampling

Biocrusts were collected in February 2018 from undisturbed vegetation sites approximately 400 km northeast of Perth in the Midwest region of Western Australia (29°13′ 1′′ S, 116°41′ 13′′ E). The regional climate is classified as Mediterranean semi-arid and receives 289 mm of average precipitation annually. Average annual surface air temperatures range from 13°C to 27°C but can attain maxima of 55–60°C in the summer months. Soils were typically red sands and the composition of native vegetation resembled those of Eremaean sclerophyll shrubland (Beard, 1990), predominated by genera such as *Acacia*, *Eucalyptus*, *Callitris*, and *Melaleuca* (Markey and Dillon, 2008).

**FIGURE 1:**
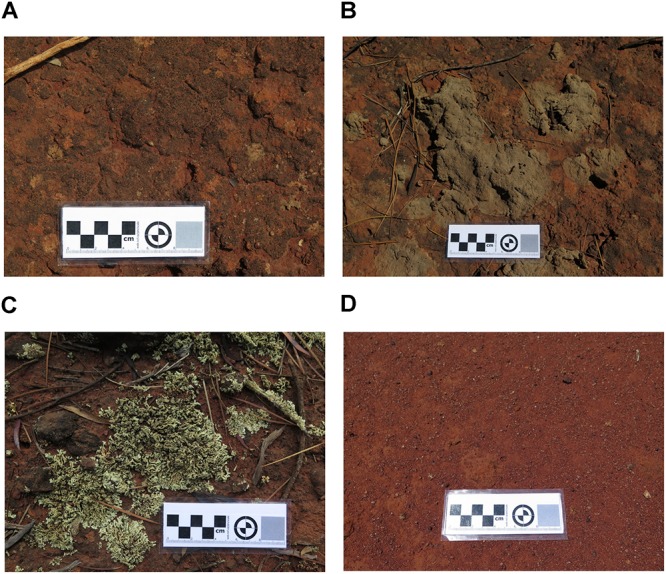
Image of sampled biocrusts, comprising of **(A)** Cyanobacterial crust, **(B)** Crustose lichen), **(C)** Foliose lichen, and **(D)** bare soil (control) sampled from an undisturbed vegetated site in Western Australia.

From two vegetated sites in proximity to a mining site, three types of biocrusts were identified visually and sampled. Cyanobacterial crust was a thin, black layer adhering to the soil with a lack of defined structure (Figure 1A). Crustose and foliose lichens (Figures 1B,C, respectively) were characterized by a better-defined structure and were easily separated from the underneath topsoil substrate. To sample biocrusts, the top layer (roughly 1 cm) was removed using a sterilized scraper (hereafter referred to as “top” samples). Bare soil (i.e., topsoil without biocrust on soil surface) was sampled to act as the control (Figure 1D). In addition, we also sampled the soil underlying biocrusts up to a depth of 10 cm (hereafter referred to as “bottom”’ samples). Underlying soil was also taken for the bare soil. At least six biological replicates of biocrust and soil, each at least 5 m apart, were collected for each crust type to provide a proper representation of habitats and landforms across the sampling site. Samples were stored at 4°C immediately after sampling and a subsample removed and stored at −20°C prior to DNA extraction.

### Chemical Analysis

Analytical chemistry was undertaken for sampled biocrusts and soils (15 g), following the methods of Rayment and Lyons (2011). Samples were air-dried and sieved to 2 mm fractions prior to chemical analysis. Sample EC and pH were measured in a 1:5 soil-water extract. Available ammonium (NH_4_^+^-N) and nitrate (NO_3_^–^-N) were extracted using 0.5 M potassium sulfate and measured by flow injection analysis (Lachat Instruments, United States). Available aluminium (Al), boron (B), calcium (Ca), cadmium (Cd), cobalt (Co), copper (Cu), iron (Fe), potassium (K), magnesium (Mg), manganese (Mn), molybdenum (Mo), sodium (Na), phosphorus (P), sulfur (S), and zinc (Zn) were extracted using the Mehlich-3 extraction and analyzed by inductively coupled plasma – optical emission spectroscopy (ICP-OES).

### DNA Extraction, 16S rRNA Gene Amplification and Sequencing

DNA was extracted from 0.25 g of biocrust and soil sample using the PowerSoil DNA Isolation Kit (MO BIO, United States). Samples were extracted in triplicate and pooled to avoid extraction bias. The 16S rRNA gene (V4 region) of extracted DNA samples were amplified using universal primers 505F (5′-GTGCCAGCMGCCGCGGTAA-3′) and 806R (5′-GGACTACHVGGGTWTCTAAT-3′) (0.2 μM), supplemented with a unique multiplexing Golay barcoded forward primer 515F in each reaction (0.2 μM), 600 ng BSA (Sigma-Aldrich, United States) and 2.5 × 5Prime Hot Master Mix (Fisher Biotec, Australia). Thermal cycling conditions were: 94°C for 45 s, annealing at 53°C for 60 s, elongation at 72°C for 90 s and final extension at 72°C for 10 min.

Amplicons were assessed visually using gel electrophoresis, using a 1.5% w/v agrose gel and subsequently purified using AMPure XP (Beckman Coulter, Inc., Australia). Purified amplicons were then quantified using DNA fluorimetry on an EnSight Multimode Plate Reader (PerkinElmer, Australia). Then, each barcoded sample mixed together to form a single pool of samples at equimolar concentrations of 40 ng prior to sequencing on an Ion Torrent PGM platform (Thermo Fisher Scientific, Australia).

### Natural δ^15^N Abundance

Approximately 50 mg of finely ground biocrust and soil samples were oven-dried and sent to the Western Australia Biogeochemistry Center^1^ for determination of total N and organic C content (referred to as total C due to the lack of carbonates within the sample). Natural ^15^N abundance was determined using a continuous flow system consisting of a Thermo Flash 1112 elemental analyser (EA) connected via a ConFlo IV interface to a Delta V Plus isotope ratio mass spectrometer (Thermo-Finnigan, Germany). Isotopic signatures (δ^15^N) were calculated using the following formula:

$$\delta^{15}\,\text{N}\;\left(\text{‰},\mathrm{Air}\right)=\left(R_{\mathrm{sample}}/R_{\mathrm{atmosphere}}-1\right)\times 1000$$

Where R_sample_ and R_atmosphere_ are the ratio of ^15^N to ^14^N of the sample analyzed and atmospheric N_2_ gas, respectively. Normalization was performed using international standards N1, N2, and USGS32 from International Atomic Energy Agency^2^.

### Sequence Analysis

Sequence data was analyzed using the QIIME (ver. 1.9) bioinformatics pipeline (Caporaso et al., 2010). Quality control parameters included minimum average quality score of 20, minimum sequence length of 130 b.p., maximum sequence length of 350 b.p., maximum length of homopolymers of 15 and maximum number of ambiguous bases of six. Strict filtering of sequence quality included single base mismatches in forward or reverse primers and barcodes causing sequence removal prior to downstream analysis. For the remaining quality filtered sequences, USEARCH (ver. 6.1) was used to remove chimeric sequences (Edgar, 2010). Operational taxonomic units (OTUs) were defined based upon sequences that shared 97% or higher identity and were taxonomically identified using the RDP classifier (Wang et al., 2007) against the Greengenes database (ver. 13.8; DeSantis et al., 2006) using UCLUST. Mitochondrial and chloroplast-like OTUs accounted for 18.28 ± 9.49 and 9.14 ± 7.27 reads percent across the entire dataset and were removed prior statistical analysis. Representative OTUs classified within the phylum *Cyanobacteria* were manually retrieved in order to place them phylogenetically into a recent and comprehensive reference dataset [published by Hug et al. (2016)]. Briefly, the reference tree was generated aligning the sequences presented in Hug et al. (2016) using INFERNAL (Nawrocki and Eddy, 2013) and the respective tree was deduced using the RAxML algorithm (Stamatakis, 2006). Query sequences (i.e., Cyanobacterial rep-set) were grafted to this reference tree using pplacer (Matsen et al., 2010) and visualized through the interactive tree of life online tool (ITOL) (Letunic and Bork, 2011) and Inkscape (ver. 0.92). All sequences have been deposited within ENA archive under project PRJEB30054.

### Statistical Analysis

Statistical analysis was undertaken on the relative frequency table constructed from a rarefied biom table (5,400 reads per sample) at family-level with richness, evenness and Shannon-Weiner diversity index calculated using the “vegan” package (Oksanen et al., 2018) within the R statistical environment (R Core Team, 2018).

Chemical properties and δ^15^N measurements were individually transformed using either log10 or Box-Cox power transformation to achieve normality and homoscedasticity prior to statistical analyses. Two-factor analysis of variance (ANOVA) was used to compare variables between crust types and depths. Relationships between measures of N cycling (e.g., δ^15^N) and various N chemical forms (total N, NH_4_-N, NO_x_-N) were explored using linear regression analysis. Plots and heatmaps were produced using “ggplot2” (Wickham, 2009) and “pheatmap” (Kolde, 2018) in the R statistical environment (R Core Team, 2018), respectively.

Euclidean distance-based principal coordinate analysis (PCoA) was applied to normalized chemical parameters, while non-metric multidimensional scaling (nMDS) was applied to 16S rRNA community data using Bray–Curtis dissimilarity (Bray and Curtis, 1957). Statistical significance of dissimilarity based on crust type and depth was assessed using main effect and pairwise permutational multivariate ANOVA (PERMANOVA). Significant chemical variables explaining microbial composition were identified using distance-based redundancy analysis (dbRDA), coupled with BEST analysis using the Spearman rank correlation. All multivariate analyses were performed using PRIMER-E v6 (Clarke and Gorley, 2006).

## Results

### Microbial Community Relationships Between Biocrusts and Soil

**FIGURE 2:**
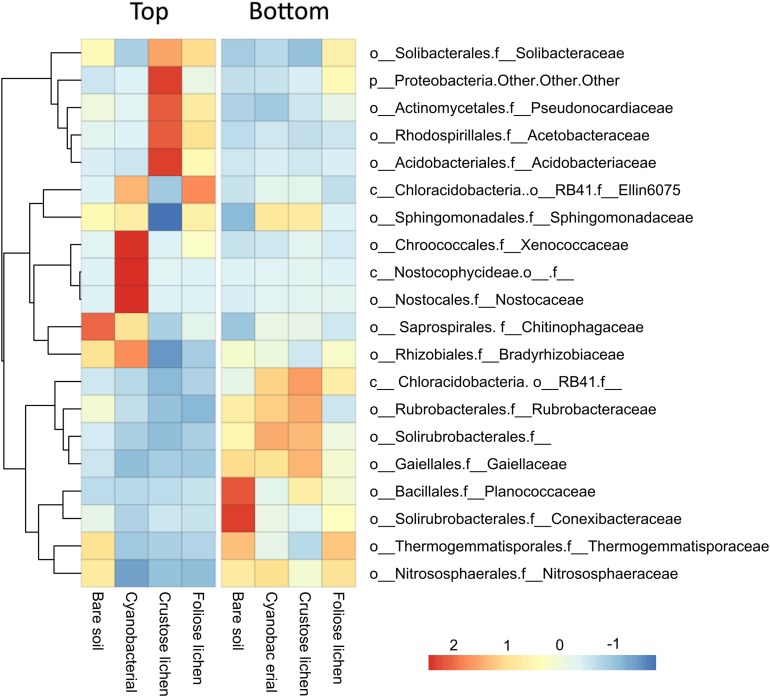
Mean relative abundance of the 20 most dominant microbial families identified in Top (bare soil and biocrust) and Bottom (underlying soil) layer sampled from an undisturbed vegetated site in Western Australia.

We retrieved a total of 2,636,503 raw sequences from 48 samples. After quality control and chimeric filtering, 406,553 high quality sequences were clustered into 19,912 OTUs. At a family level, microbial communities in biocrusts (column “Top”) were compositionally distinct from those found within the underlying soil (column “Bottom”) for the 20 abundant most dominant families (Figure 2). Microbial communities within cyanobacterial crust were highly correlated with *Rhizobiales* (phylum *Proteobacteria*), *Chroococcales*, *Nostocales* one unknown family within the class *Nostocophycideae*, all belonging to the phylum *Cyanobacteria*. Crustose lichen, and to a lesser extent, foliose lichen crusts were dominated by OTUs falling within the order *Acidobacteriales* (phylum *Acidobacteria*), *Rhodospirillales* (phylum *Proteobacteria*), and *Actinomycetales* (phylum *Actinobacteria*). Crustose lichen alone also harbors high relative abundance of unclassified OTUs within the phyla *Proteobacteria*. Microbial communities within the underneath topsoil were dominated by more even mix of Actinobacterias (i.e., orders: *Rubrobacterales*, *Solirubrorales*, and *Gaiellales*), alongside *Bacillales* (phyla: *Firmicutes*), *Thermogemmatisporales* (phyla: *Chloroflexi*), and *Nitrososphaerales* (phyla *Crenarchaeota*). Archaeal taxa were detected in our samples, but at a low abundance (<1% in all crust types), whilst the soil beneath contained a higher proportion (2.43–3.97%). In all samples, the dominant family was *Nitrososphaeraceae*, which was more abundant in soils (3.31 ± 0.21%) when compared to biocrusts (0.66 ± 0.01%). However, the abundance of *Nitrososphaeraceae* was likely linked to the soil substrate itself, as they were also abundant in the top layer of bare soil.

**FIGURE 3:**
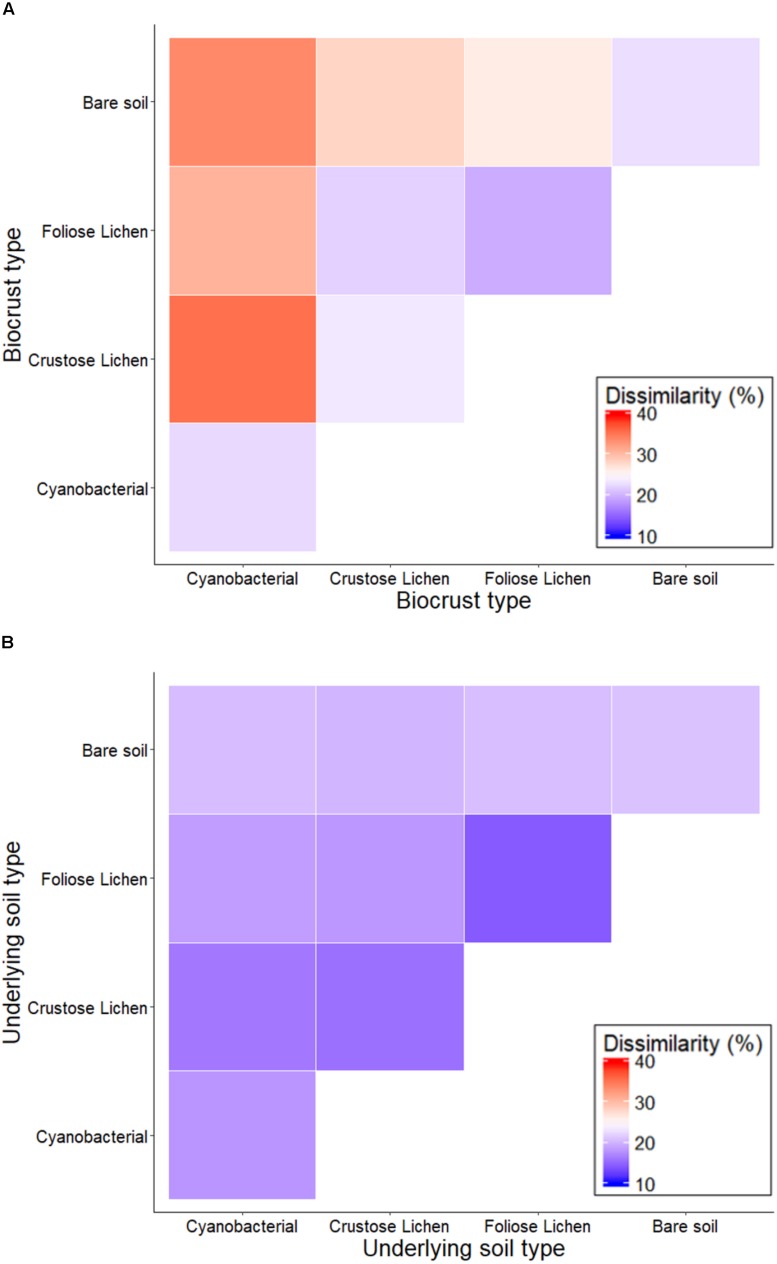
Percentage dissimilarity when comparing family-level intra- and inter-crust microbial communities in **(A)** Top (bare soil and biocrust) and **(B)** Bottom (underlying soil) layer sampled from an undisturbed vegetated site in Western Australia.

Non-metric multidimensional scaling of family-level 16S rRNA gene identified taxa revealed distinct clusters when classified according to crust type and depth (data not shown), distinguishing biocrusts between crust type and from the soil beneath them, which was also reflected in pairwise dissimilarity matrices (Figure 3). PERMANOVA analysis of community structure of biocrust and soil further supported differentiation in crust types (*F* = 4.522; *p* = 0.001), depth (*F* = 18.18; *p* = 0.001), and their interaction (*F* = 3.159; *p* = 0.001). Intuitively, within biocrust dissimilarities (Figure 3A) were higher than within underneath soils (Figure 3B).

**FIGURE 4:**
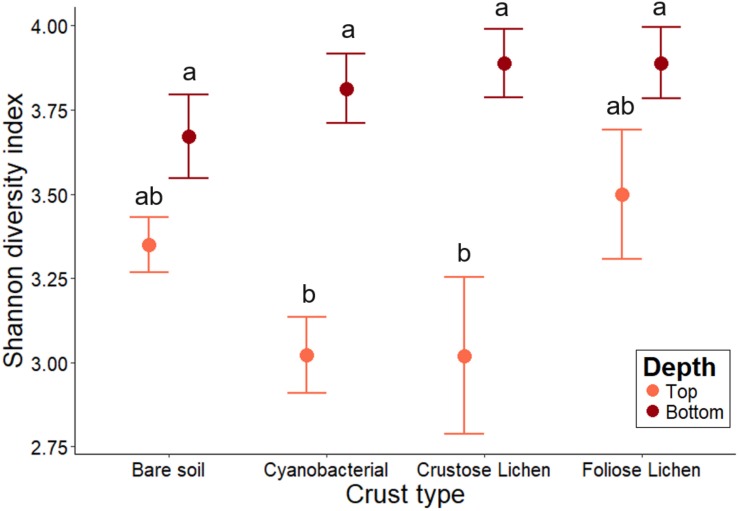
Family-level microbial community Shannon-Weiner diversity in Top (bare soil and biocrust; orange fill) and Bottom (underlying soil; brown fill) layer sampled from an undisturbed vegetated site in Western Australia. Error bars indicate one standard error of the mean. Annotated letters indicate statistical significance (*p* < 0.05) between crust type and depth.

Shannon-Weiner diversity indices ranged from 2.56 to 4.30, with higher diversity found in the soils beneath the biocrusts (Figure 4). This suggests selection within the biocrusts relative to the soil beneath, which generated a reduced microbial diversity (Figure 4). Diversity indices between crust types were broadly similar (*p* > 0.2) and were consistently lower than in the underlying soil layer, though this was not significant for Foliose crust and its underlying soil (*p* > 0.4). In the bare soil, diversity between the top surface layer and deeper soil layer was also similar (*p* > 0.6). The microbial diversities of cyanobacterial and crustose lichen were consistently lower than their underlying soil (20.7 and 22.3%, respectively; *p* = 0.001, 0.002), likely due to strong selection pressures within crusts themselves, which is possibly linked to the age or developmental stage of these biocrusts.

### Nitrogen Cycling Capability Between Biocrusts and Soil

**FIGURE 5:**
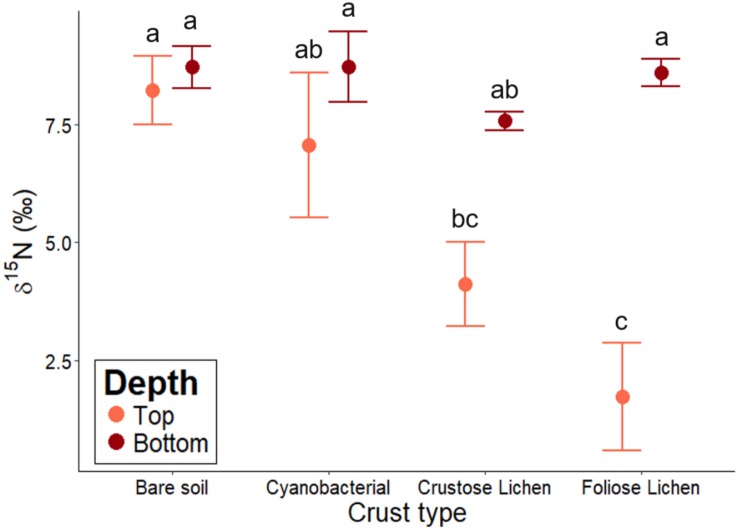
δ^15^N abundance of different biocrust types, bare soil and their underlying soil sampled from undisturbed, vegetated areas in the Midwest region of Western Australia. Error bars indicate one standard error of the mean. Annotated letters indicate statistical significance (*p* < 0.05) between crust type and depth.

Biocrusts’ δ^15^N ranged from 1.72 to 7.6 and followed a decreasing trend in the following order: bare soil > Cyanobacterial crust > Crustose lichen > Foliose lichen (Figure 5). However, Cyanobacterial was not significantly lower than the bare soil (*p* = 0.96), and Foliose lichen was not significantly lower than their Crustose counterpart (*p* = 0.78). Soil δ^15^N did not change significantly across crust types, ranging from 7.58 to 8.73 (*p* > 0.5). Only δ^15^N in Foliose lichen was significantly different from its underlying soil (*p* = 0.001).

### Chemical Properties and Their Relationship With Microbial Communities and N-Fixation

**FIGURE 6:**
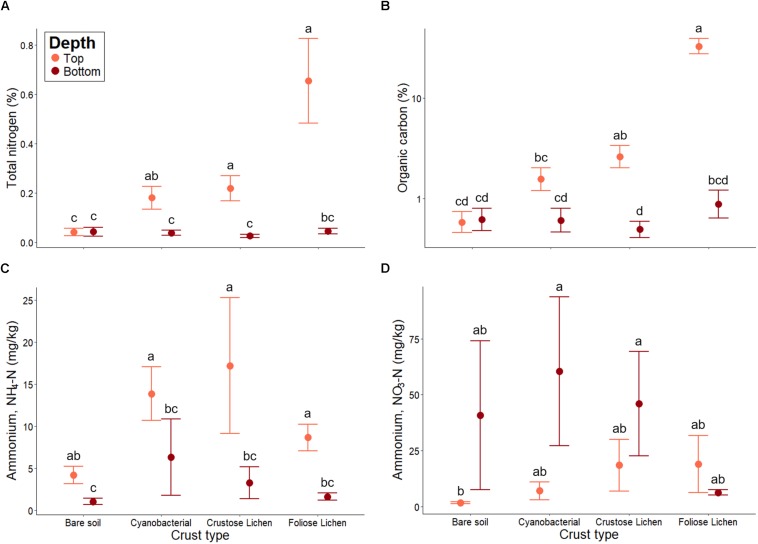
**(A)** Total nitrogen, **(B)** total organic carbon, **(C)** extractable ammonium (NH_4_-N) and **(D)** extractable nitrate (NO_3_-N) concentrations in Top (bare soil and biocrust; orange fill) and Bottom (underlying soil; brown fill) layer sampled from an undisturbed vegetated site in Western Australia. Error bars indicate one standard error of the mean. Annotated letters indicate statistical significance (*p* < 0.05) between crust type and depth.

Total N was significantly higher in all crust types when compared to bare soil (*p* < 0.01; Figure 6A). Foliose lichen exhibited the most elevated N concentration (15.9 times higher than bare soil) and Cyanobacterial crust the lowest (4.39 times). Total N was also higher within biocrusts, compared to the underlying soil (*p* < 0.01). A similar trend was observed for total organic C (Figure 6B). Notably, total N and C in Foliose lichen were 16 and 50 times greater than bare soil, respectively (*p* < 0.001).

Higher concentrations of NH_4_^+^-N were consistently found in biocrusts, compared to their underlying soil (*p* < 0.001; Figure 6C). Bare soil also contained more NH_4_^+^-N within its top layer (*p* < 0.001), though this difference was not as great as in biocrust-underlying soil comparisons. Furthermore, NH_4_^+^-N did not differ among crust types, nor did they differ from the bare soil (*p* > 0.01). Conversely, NO_3_^–^-N concentrations were consistently lower in biocrusts and bare soil, compared to their underlying soil (Figure 6D). However, NO_3_^–^-N within the underlying soil were highly variable and were not significantly higher than those in the biocrust (*p* > 0.1). Nevertheless, we found that NO_3_^–^-N increased incrementally from the bare soil to Foliose lichen in the top layer, consistent with trends observed in total N and organic C.

Similar to 16S rRNA community composition, we also found differentiation between crust type and depth in terms of the chemical composition (data not shown). Pairwise PERMANOVA also suggested significant dissimilarities between crust types (*p* < 0.034), and significant similarities between their underlying soils (*p* > 0.2). In order to link soil chemistry and 16S rRNA gene profiles, dbRDA was conducted to identify significant chemical parameters that explained family-level microbial community composition (Supplementary Figure S1). Along the primary axis, which discriminated against biocrust and soil, total N, NH_4_^+^-N, P, organic C and Zn strongly correlated with biocrusts. The secondary axis showed that positive correlation of pH and Cd differentiated Cyanobacterial crust communities from those in Crustose and Foliose crust. BEST analysis revealed that δ^15^N was the single strongest predictor for community composition (ρ = 0.40), followed by NH_4_^+^-N, Cd, Al, and total N (Supplementary Table S1). Collectively, they improved correlation between microbial community and chemical composition (ρ = 0.57). The variation in δ^15^N was best explained by available Fe (*R*^2^ = 0.54) and organic C (*R*^2^ = 0.52).

## Discussion

### The Microbial Composition of Biocrusts Is Unique to Different Crust Types and Differs From the Surrounding Soil Environment

By employing a combination of marker gene sequencing and stable isotopic techniques, we demonstrated that strong distinctions within the bacterial microbiome communities of biocrusts, both between the crust types themselves and with the soil beneath them. We also demonstrated wide variations in genetic capacity to fix atmospheric N and relative N-cycling quantities between these biocrusts, as well as examine the potential transfer of these processes to the biocrusts’ soil surroundings. We believe this is the first comprehensive microbial phylogenetic and functional analysis performed to simultaneously compare various biocrust types to their surrounding and underlying soil environment. Our findings shed significant new light upon the structure and function of these enigmatic ecosystems, especially in the Western Australian landscape, where its contribution to the overall ecosystem health has been systematically underrated when restoring disturbed landscapes.

When assessing the soil microbial communities within the study area, we determined that they were comprised primarily of *Actinobacteria*, *Acidobacteria*, *Bacteroidetes*, *Proteobacteria*, and *Chloroflexi* taxa (Figure 2). These findings are consistent with those from other semi-arid environments, particularly the dominance of *Actinobacteria* (Chanal et al., 2006; Connon et al., 2007; Kutovaya et al., 2015). *Rubrobacteraceae* was the most abundant family of *Actinobacteria* detected, again, similar to an earlier survey of arid Australian soil (Holmes et al., 2000). Makhalanyane et al. (2015) noted that this is perhaps due to their wide metabolic capacity, which includes UV repair and secondary compound synthesis. The dominance of the archaeal *Crenarchaeota* have also been observed previously (Chanal et al., 2006; Kutovaya et al., 2015).

In sharp contrast, bacterial communities in native biocrusts are different from those found in bare soil. While *Actinobacteria* remained abundant in biocrusts, it was not the most abundant phylum and their dominance varied by biocrust type. In Cyanobacterial crust, *Cyanobacteria* were the most abundant phylum, which was in line with other biocrust-related studies (Nagy et al., 2005; Strauss et al., 2012; Steven et al., 2013). Most notably, we failed to taxonomically classify any OTUs as *Microcoleus*, a free-living cyanobacterial genus often reported in biocrusts in the United States (Yeager et al., 2004; Nagy et al., 2005; Steven et al., 2013), Spain (Maier et al., 2014), and South Africa (Maier et al., 2018). Chilton et al. (2017) also failed to detect OTUs related to this genus in biocrusts in a semi-arid region of eastern Australia. Belnap (2002) noted that as crusts mature, *Microcoleus* becomes replaced with other cyanobacterial genera such as *Nostoc* and *Scytonema*, which were indeed abundant in Cyanobacterial crust. These genera are responsible for the production of scytonemin, a metabolite that filters out damaging UV radiation and gives these types of biocrust a dark pigment (Castenholz and Garcia-Pichel, 2000), which might give the former an important ecological advantage in semi-arid environments.

**FIGURE 7:**
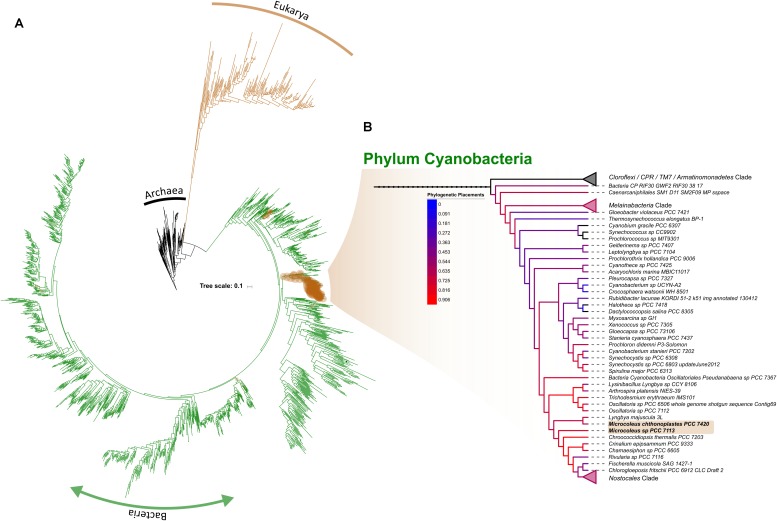
Phylogenetic placement of OTUs identified as within the phylum Cyanobacteria across the entire dataset. Brown circles correspond to the position where the placement is suggested while it sizes correlates with the amount of OTUs per site **(A)**. Subtree showing the Cyanobacterial branch, with branch color representing the amount of OTUs placed within **(B)**. For simplicity, collapsed nodes were selected by their absent (top-most; Cloroflexi, CPR, TM, and Armatinomonadetes phyla) or low abundance of placements (phylum Melainabacteria and order Nostocales).

As reference datasets usually lack the ability to fully resolve lower taxonomic levels (Kozlov et al., 2016), we retrieve all OTUs classified within the phylum Cyanobacteria in order to placed them, phylogenetically, into a comprehensive reference tree recently published by Hug et al. (2016). While taxonomy-based results showed that cyanobacterial OTUs were mainly comprised of the orders *Oscillatoriales* and *Nostocales*, with a smaller proportion of *Synechococcophycideae*, which agrees with similar study in the Pilbara region of Western Australia (Muñoz-Rojas et al., 2018); a phylogenetic placement of cyanobacterial sequences shows a well scattered read placement across the entire cyanobacterial branch (Figure 7A). Besides *Oscillatoriales* and *Nostocophycideae* classes previously resolved with a taxonomic approach, we further identified OTUs falling within *Pleurocapsales*, *Chroococcales*, and *Prochlorales* (Figure 7B). More importantly, *Microcoleus*-like reads were identified through this method, accounting for 28.53% of all the cyanobacterial representative sequences (available in https://doi.org/10.6084/m9.figshare.8480459.v1). The discrepancy between taxonomic/phylogenetic approaches suggest that re-analysis of published cyanobacterial data originated from this region’s biocrusts is highly suggested in order to fully identify the cyanobacterial species spectrum inhabiting them.

Regardless of this discrepancy, a less dominance of *Microcoleus* in WA biocrust suggest that two possible drivers shaping the cyanobacterial community within them: the first being that biocrusts mature through a development gradient toward more resistant cyanobacterial taxa due to UV exposure, or, that the biological role of *Microcoleus* may be fulfilled by different cyanobacterial taxa in Western Australian arid biocrusts (Chilton et al., 2017). A repeated sampling defined-age biocrusts is required to resolve either of these drivers. Aside from *Cyanobacteria*, we also detected abundant *Chloracidobacteria*, which is the only *Acidobacteria* capable of photosynthesizing (Bryant et al., 2007), suggesting that C-fixation pathways within the biocrust may not be driven solely by their *Cyanobacteria*, as previously thought.

### Interactions Between Different Lichens and Microbiome, Together With the Chemical Environment Within Biocrust, Drive Differentiation in Microbial Communities

In Crustose and Foliose lichen crust, *Proteobacteria* and *Acidobacteria* were equally dominant phyla. The prevalence of *Proteobacteria*, specifically *Alphaproteobacteria*, found in lichen-dominated crusts has been widely reported (Bates et al., 2011; Maier et al., 2014; Grube et al., 2015). Studies that employed fluorescence *in situ* hybridization supported these findings and further demonstrated that the distribution of *Alphaproteobacteria* within the lichen structure varied by crust type (Grube et al., 2009) and species (Maier et al., 2014). However, the relatively high abundance of *Acidobacteria* within the two lichen biocrusts is more unusual and may be unique to the environment studied, suggesting endemism may occur within the biocrust’ microbial communities when examining local populations at large scales. One explanation offered by Hodkinson and Lutzoni (2009) and Bates et al. (2011) inferred that substantial amounts of organic acid secondary metabolites produced by lichens could lead to an environment that favors the growth of acid tolerant *Acidobacteria* and hence, a possible mechanism of strong selection for these taxa observed within lichen-dominated biocrusts. Interestingly, we also determined that whilst both lichen-dominated crusts were similar in terms of microbial composition at a phylum level, a significant and consistent divergence was observed at lower taxonomic levels (i.e., family). Similarly, Grube et al. (2009) and Bates et al. (2011) showed that microbial communities of these biocrusts differed more by lichen species, rather than other factors such as spatial proximity. Our findings similarly suggest that lichen-associated microbial communities are highly structured and likely as a result of lichen-microbe selection and interaction.

When examining chemical properties of the biocrusts, it was clear that the chemical environment within each crust type were unique and explained a significant proportion of variation in microbial composition. Therefore, we suggest that the unique geochemical composition found within each biocrust is a significant driver of selection for specific microbial communities. For example, microbial community patterns in the lichen-dominated crusts were negatively correlated with pH, which favored populations of *Acidobacteria*.

Overall, N was also an important variable in predicting microbial community patterns, as δ^15^N, NH_4_^+^-N, and total N were well-correlated with microbial composition, suggesting that N-cycling processes within the biocrusts differ from the soil around them and impacts the resultant biocrust microbiome.

### Biocrusts Exhibit Highly Selected Communities With Lower Diversity Than the Surrounding Soils

For diversity metrics within the crusts, it was clear that species richness within the biocrusts was different from bare soil, as were taxa composition. Overall, Shannon indices were lower in biocrusts when compared to the underlying soil, suggesting again, that strong selection takes place within the crust. Steven et al. (2014) suggested that low diversity in cyanobacterial crusts was likely due to dominance of cyanobacterial taxa, resulting in skewed community composition. For biocrusts in general, Mueller et al. (2015) suggested that below-crust soils were less exposed to UV and moisture stress whilst biocrusts experienced high levels of these abiotic stresses and thus, faced strong selection pressures. These factors would explain our observations of reduced diversity within the crusts due to strong selection of those organisms, which can survive harsh arid environments with high UV indices.

### Nitrogen Cycling Capability Within Different Biocrust Types

Based upon δ^15^N data, N-fixation was most prevalent in Foliose lichen crust. The δ^15^N values fell within the range of −2 and +2‰, indicating its N was largely derived from the atmosphere (Shearer and Kohl, 1986). Our findings support those of Pate et al. (1998), where they found that N-fixation was likely driven by cyanobacterial lichens, rather than leguminous trees and shrubs, at a site in the same region. We further found available Fe and organic C to be the best predictors of δ^15^N, we assume due to the fact that N-fixation is an energetically expensive process and requires available Fe to synthesize nitrogenase, the key enzymes that catalyze N-fixation (Reed et al., 2011).

The δ^15^N of Cyanobacerial crust and Crustose lichen were higher than expected, as previous studies found Cyanobacterial (Evans and Belnap, 1999; Billings et al., 2003) and lichen (Heindel et al., 2018) biocrusts falling within the −2 and +2‰ range in their natural environment. However, due to their morphologies, these crust types may have contained soil material adhering to it when we sampled, subsequently affecting their δ^15^N values. Conversely, Foliose lichen had a foliar structure and was easier to separate from the soil and clean. Another potential explanation is the prevalence of N-loss processes (e.g., incomplete nitrification, denitrification) in Cyanobacterial and Crustose types, which can result in relative ^15^N enrichment (Barger et al., 2016). Furthermore, dark cyanobacterial crusts were found to emit nitric oxide and nitrous oxide (Barger et al., 2005; Abed et al., 2013), particularly under hot conditions (i.e., summer period; Weber et al., 2015). Potentially lower δ^15^N may be derived from biocrusts when sampled during winter months, where N-fixation activity increases, and N-loss processes decreases (Barger et al., 2005).

Nevertheless, the strong relationship between δ^15^N and total N suggests that N-fixation can potentially result in the enrichment of soil N stock. Despite δ^15^N of Cyanobacterial and Crustose lichen being outside the described range, they were still closer to atmospheric N relative to the bare soil, corresponding to higher total N content, which was reported across a range of crust types and ecosystems (Evans and Ehleringer, 1993; Billings et al., 2003; Deane-Coe and Sparks, 2016). While our δ^15^N data points to the N-fixing potential of biocrusts, further investigation using isotopic tracer techniques is required to properly quantify the magnitude of this effect.

### Effect of Crust Type on Biocrust and Underlying Soil Chemistry

The positive effects of biocrusts upon surface C and N accumulation have been demonstrated in different ecosystems (Beraldi-Campesi et al., 2009; Brankatschk et al., 2012; Maier et al., 2014; Heindel et al., 2018). Here, we found that C and N content in biocrusts varied by crust type, where lichen-type crusts contained higher total N and organic C. However, we found that the presence of biocrust had limited influence on the N content of their underlying soil. This was reported in other biocrust studies in cold sandy dunes (Brankatschk et al., 2012) and both cold (e.g., Colorado Plateau; Barger et al., 2005) and warm (e.g., Sonoran Desert; Beraldi-Campesi et al., 2009) desert environments. Similar to our findings, a temporal study by Castillo-Monroy et al. (2010) observed no significant difference in NH_4_-N between biocrust and bare soil. Furthermore, they also reported that NO_3_-N concentration was higher in bare soil compared to biocrust, though not significantly.

Additionally, the positive impacts of biocrust on underlying soil nutrients have also been documented. In a sandy dune system, Guo et al. (2008) detected higher organic matter, total N and available N in soils underneath biocrusts, compared to non-crusted soil. Though leaching of available N from biocrusts has been clearly demonstrated (Thiet et al., 2005; Johnson et al., 2007), this has not been uniformly observed. As such, the fate of available biocrust N remains a controversial topic (Belnap, 2002; Johnson et al., 2007; Barger et al., 2016). We postulate that biocrusts do influence their underlying soil, but this is likely limited by depth of influence. Using millimeter-scale measurements, Johnson et al. (2007) showed that soil inorganic N was highly variable even within the biocrust structure, peaking at a depth of 2 mm. Our strategy for sampling soil underlying biocrusts may thus have had a diluting effect by including soil below a zone of influence if this zone only extends a few millimeters within the soil surface. Barger et al. (2005) found no difference in NH_4_^+^-N and NO_3_^–^-N in various biocrusts when sampling at a depth of 5 cm, though this was inconsistent with a later study at the same site, where sampling depth was restricted to the top 2 cm (Barger et al., 2013). Therefore, we suggest that high resolution sampling may be required to fully confirm the capability of the crusts to act as an N source into the immediate source environment. We note that strong temporal controls exist both directly influencing mineral N production and consumption, but also its location within the soil profile due to leaching and infiltration following rainfall. Seasonality of mineral N was not the subject of this study, but should be the topic of future research efforts to understand N dynamics in these systems.

## Conclusion

There is increasing recognition for biocrust research globally due to their important ecological roles, and especially as microbial engineers for semi-arid ecosystem restoration. Characterization of the bacterial communities within these crusts coupled to relevant biogeochemical analysis can enable better understanding of their functional roles. Here, we demonstrated strong distinctions in the microbiomes of various biocrusts found in the Midwest region of Western Australia. Microbial community composition was dependent upon crust type and is likely a product of community interactions (e.g., lichen-microbe interactions), chemistry within the crust and the ability to survive extremes of aridity and UV index. Crust type also plays a role in determining apparent N-fixing potential, which has subsequent effects on their C and N content. However, biocrusts have limited influence upon their underlying soil, which did not show enrichment of N, even when directly underneath high N-fixing biocrusts. Finally, and based on taxonomic and phylogenetic results discrepancies when retrieving *Microcoleus* spp. in Cyanobacterial crust, we strongly suggest the application of the later methodology for detailed biocrust-associated microbiome descriptions, or at least, when finer classification levels are desired.

## Author Contributions

BM-G, KT, JY, and AC designed the study. KT and JY performed the field sampling, and KT and BM-G performed microbiome analyses with KT preparing samples for chemical analyses. BM-G provided bioinformatics analysis and statistical expertise and analyzed these data with KT. MF provided expertise on N-cycling. AW and DK provided facilities and expertise for microbiome analyses, while PN provided facilities and expertise in semi-arid landscape restoration. BM-G and KT led the writing of the manuscript with significant inputs from AW, AC, and MF. All authors commented and made additions to the manuscript during revision stages.

## Conflict of Interest Statement

The authors declare that the research was conducted in the absence of any commercial or financial relationships that could be construed as a potential conflict of interest.

## Footnotes

1 http://www.wabc.uwa.edu.au/

2 https://nucleus.iaea.org/rpst/index.htm
