# Supplementary material for: The Bacterial Microbiome Associated With Arid Biocrusts and the Biogeochemical Influence of Biocrusts Upon the Underlying Soil
Source: Front Microbiol. 2019 Sep 23;10:2143. doi: 10.3389/fmicb.2019.02143 (PMC6768011; doi:10.3389/fmicb.2019.02143)
Supplement: Supplementary file 1 [file Data_Sheet_1.docx]

Supplementary Material

**Supplementary Table 1.** Correlation coefficient of chemical variables best able to explain variation in family-level bacterial community composition in biocrust and soil collected from undisturbed, vegetated areas in the Midwest region of Western Australia, as revealed by Spearman correlation rank BEST analysis.

| **No. of**  **variables** | **Variables** | **Correlation coefficient, R** |
| --- | --- | --- |
| 1 | δ15N | 0.399 |
| 2 | δ15N + NH4-N | 0.484 |
| 3 | δ15N + NH4-N + Cd | 0.530 |
| 4 | δ15N + NH4-N + Cd + Al | 0.564 |
| 5 | δ15N + NH4-N + Cd + Al + Total N | 0.573 |

**
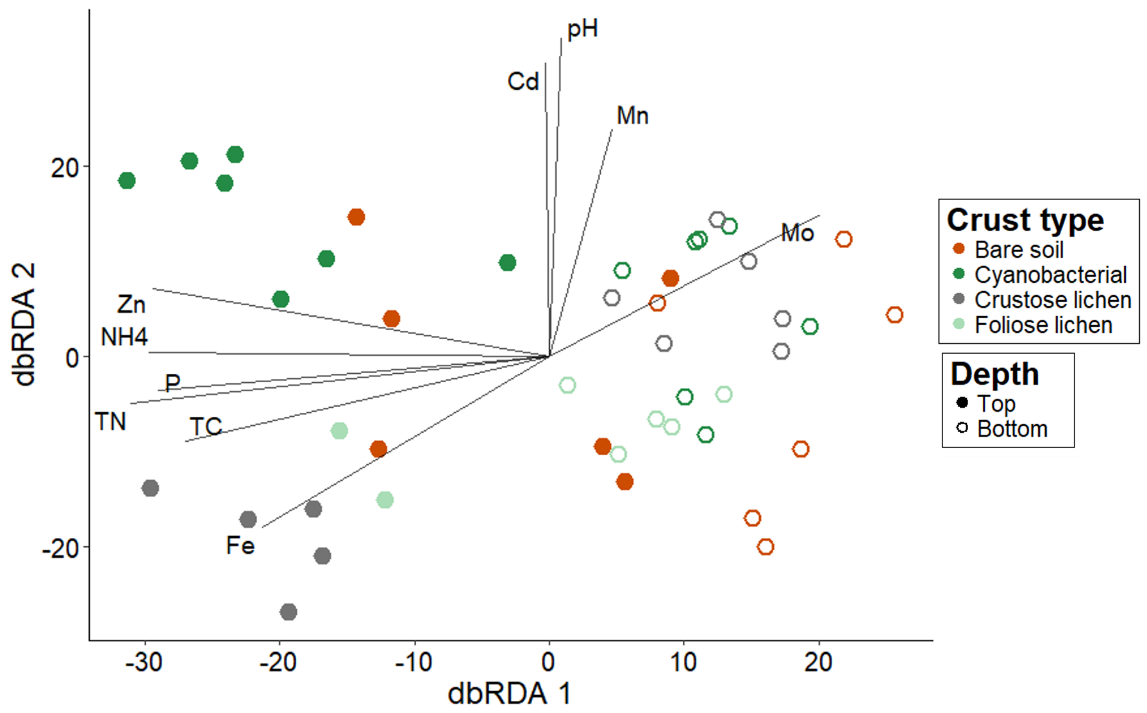
**

**Supplementary Figure 1.** Distance-based redundancy analysis (Bray-Curtis dissimilarity) of family-level bacterial communities in different biocrust types, bare soil and their underlying soil sampled from undisturbed, vegetated areas in Western Australia. Vectors indicate highly correlated chemical variables (R > 0.5 and 0.7, respectively). Colours indicate crust type.
